# Influence of Prefabricated Construction on the Mental Health of Workers: Systematic Review

**DOI:** 10.3390/ejihpe13020026

**Published:** 2023-02-01

**Authors:** Rasaki Kolawole Fagbenro, Riza Yosia Sunindijo, Chethana Illankoon, Samuel Frimpong

**Affiliations:** School of Built Environment, The University of New South Wales, Sydney, NSW 2052, Australia

**Keywords:** construction workers, health and safety, mental health, stressors, prefabricated construction

## Abstract

Despite the significant contribution of the construction industry to national and global economies, the risk it poses to the health and safety of its workers is concerning. With substantial improvement in physical health and safety performance, especially in industrialised and developed economies, attention has shifted to the mental health of construction workers. The construction industry has implemented several worker-focused and management-oriented intervention programs, but problems related to poor mental health persist, and the industry ranks high in suicide figures. Entering the Construction 4.0 era, the use of technologies and new construction methods have been touted to have the potential to improve mental wellbeing. Therefore, this research addresses this lingering problem by: (1) identifying and classifying stressors of mental health and (2) assessing the relevance of adopting prefabricated construction to improving mental health. A two-phased PRISMA-guided systematic review was conducted due to the nonavailability of past studies that combine the concepts of prefabrication and mental health. Mental health stressors were grouped into three categories, with industry-related identified as having an influence on management/organisational and personal stressors. Prefabricated construction, on the other hand, by virtue of its benefits over traditional construction, is found to be capable of eliminating, or at least reducing, the impact of industry-related stressors and, by extension, promoting good mental health.

## 1. Introduction

Construction is one of the largest industrial sectors globally and has significant contributions to the development of national and global economies. Recently, the mental health of construction workers has attracted more attention among researchers and industry stakeholders. Despite the significant improvements made in physical health and especially safety by developed countries, the construction industry still struggles with the problem of poor mental health among its workers [1,2]. This has led to a significant decline in the construction industry’s productivity due to the loss of active manpower to early retirement [3] and death through suicide [4,5]. When compared to other industries, the degree of mental health challenges among construction workers is high. In Australia, for instance, Lingard and Turner [1] affirm that the mental health record of construction workers is poorer than that of the general Australian population. Although several causal factors of the prevalent poor state of mental health among construction workers have been cited in past studies, prominent among these stressors, as they are usually called, are linked to work conditions or the nature of construction work and business, management, and personal factors [6]. There is a good number of peer-reviewed research articles on various intervention programs to curtail the effects of poor mental health in the construction industry; however, these studies and the intervention programs have focused on addressing the management [7] and personal stressors [8] neglecting those stressors that are related to the nature of the industry. The presence of these industry-related stressors influences personal and management/organisational stressors. This may explain why most of the intervention programs that are targeted at creating awareness and providing support for construction workers have not yielded satisfactory results. Making meaningful progress in the issue of mental health in the industry, therefore, involves addressing the stress-inducing nature of construction work, such as uncertainty in project duration [9], conflicts and disagreements [10], over-reliance on skilled workers [11], excessive exposure to weather conditions [12], complex design coordination requirements [13], or bodily and musculoskeletal pains [3]. To tackle the issue of mental health from its root cause, this study sets out two objectives to be met through an extensive review of the literature:

1. Identify and classify mental health stressors in accordance with their sources.
2. Assess the relevance of prefabricated construction techniques in improving the mental health of construction workers.

Although the concept of prefabricated construction is not new, the method has not been widely embraced despite its glaring benefits over the traditional in situ construction method [11,14,15]. Prefabricated construction involves the design and manufacture of building components or modules, to a greater degree of finish, in a controlled factory environment before transporting them to the construction site for final installation. Prefabrication encompasses standardisation, which is the extensive, regular, and repetitive use of methods and/or processes in manufacturing building components and/or modules. Several studies have affirmed the benefits of prefabricated construction over traditional construction, especially in terms of cost, quality, time, and health and safety performance [16]. However, no study has focused on the influence of the benefit of prefabrication on construction workers’ mental health. Although back pain in workers, arising from postural load and whole-body vibration from using vibrating equipment, has been linked with precast concrete, which is a form of prefabrication [17], later studies have addressed this drawback by proposing incorporating ergonomics into the workplace design and layout [18,19]. With a drastic reduction in workers’ exposure to working under adverse weather conditions [11,14,20], the provision of a conducive physical working environment is guaranteed. King et al. [5] indicate that the work environment can play significant roles in positively enhancing mental health and reducing suicidal behaviours among construction workers. Therefore, prefabrication through the elimination of the weather’s influence may enhance workers’ productivity and, consequently, reduces working hours, promotes more relaxation time for workers, reduces site incident and injury occurrence, and generally, presents a better conducive working environment that eliminates stressors that are primarily responsible for poor mental health among construction workers which are described as work-related by Pidd, et al. [21].

Furthermore, the choice of using any construction method is usually based on cost, quality, and time performance, as these parameters, are strongly tied to value for investment by the project client [22]. As such, attention is shifting towards prefabrication as a means of adequately responding to the increasing demand for construction projects, because of its potential to deliver projects faster than the traditional method. In addition to direct cost benefits, prefabricated construction could positively influence the mental health of workers and thereby reduce cases of early retirement and suicide. Addressing work-related stressors or factors of construction that pose cultural and organisational barriers to pursuing a healthy lifestyle by construction workers is expected to be more effective in promoting good mental health [1]. With prefabrication potentially enhancing positive work–life balance through faster construction [9] among other benefits, the implication is expected to, in addition to improving workers’ mental health, improve the industry’s productivity and promote a better standard of living among the workers.

## 2. Materials and Methods

The objectives of this study informed the search criteria and keywords used in the literature search. The search for mental health and prefabricated construction keywords and related terms did not return any article that is based on the two phenomena. Therefore, the literature searches were conducted separately, with one focusing on the mental health of construction workers and the other exploring the general health and safety benefits of prefabricated construction.

### 2.1. Phase 1—Mental Health Stressors in Construction

The first phase of the search was focused on identifying stressors of mental health among construction workers and the intervention programs that have been proposed and implemented.

#### 2.1.1. Literature Search Strategies and Terms

Three electronic databases, Scopus, Web of Science (WOS), and PubMed, were searched for the study keywords with combination strings (“AND” and “OR”) as appropriate. The Scopus and Web of Science databases were selected because of their wide coverage in different types of research publications in sciences, engineering, construction, and other fields of research [23,24]. Since the study is on health-related issues in the construction industry, PubMed was also selected because of its reliability as the largest repository of health-related research [25]. The search was limited to publications in the English language and/or articles with English interpretation of the original language of publication. No restriction was placed on either country of focus or the date of publication to have a feel of the nature of the phenomenon in different geographical locations.

The keywords for this phase of the search were combined with the Boolean strings “AND” and “OR”) as: (“mental health” OR “mental illness” OR “mental ill-health” OR “mental disorder” OR “anxiety” OR “anxiety disorder” OR “depressive disorder” OR “depression” OR “schizophrenia” OR “substance use” OR “substance abuse” OR “drug abuse” OR “bipolar disorder” OR “mental well-being” OR “mental wellbeing” OR “psycholog*”) AND (“construction worker*” OR “construction professional*” OR “construction tradespe*” OR “construction artisan*” OR “construction industr*” OR “aec” OR “architecture engineering and construction” OR “building construction” OR “construction organi*” OR “construction firm*”). The research team, which includes experts in the field of construction health and safety management research and with practical experience in the construction industry, ensured that the keyword list was comprehensive and contained terms that are established in the literature. In addition, past review studies [26,27,28,29] on construction workers’ mental health were consulted to confirm the comprehensiveness and adequacy of the selected keywords. The keywords were carefully selected to include possible alternate references for the study focus: mental health and the construction industry. A total of 2679 articles were obtained from the databases with Scopus having the highest result with 1252 followed by Web of Science with 876 and PubMed with 551.

#### 2.1.2. Exclusion and Inclusion Criteria

Exclusion and inclusion criteria set for eligibility to form a part of the review materials were set. Only the articles that met all the following inclusion criteria were included in the review:

1. Articles that studied either the causes of or intervention for mental health among construction workers;
2. Articles that studied common mental health disorders (such as anxiety, depression, behavioural disorders, and posttraumatic stress disorder) among construction workers of all ages, gender, occupations, professions, and cadres;
3. Conducted the study and made decisions based on empirical data collected. Where pilot studies were considered, such studies must have conducted full-scale data collection and included in the whole analysis and findings;
4. Studies based on scoping review, systematic review, and any other forms of reviews of the literature were excluded;
5. Articles must have been published in English. Articles published in other languages other than English but with official English versions available were included;
6. Articles published in peer-reviewed journals were included. Other articles, such as conference papers, book chapters, reviews, white papers, etc., were excluded even in cases where they met other inclusion requirements. Apart from the fact that journal publications undergo a stringent peer-review process, it is widely confirmed among researchers that they are the most reliable and comprehensive source of knowledge in a research area.

#### 2.1.3. Data Extraction and Final Article Selection

**Figure 1:**
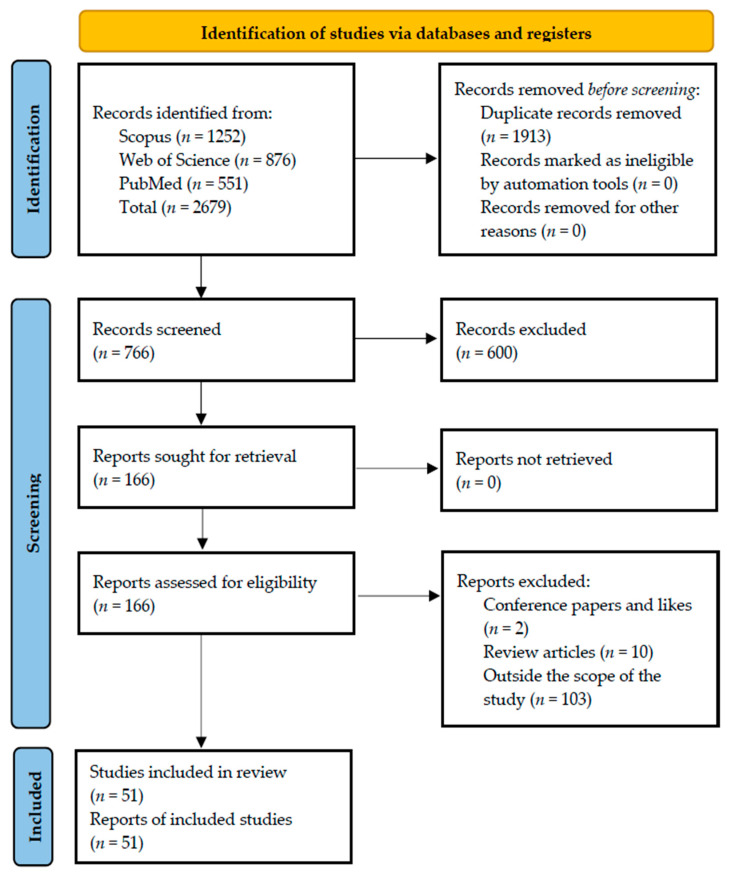
Flowchart of the literature search and selection strategy for the mental health of construction workers.

The articles were uploaded on https://www.rayyan.ai/ (accessed on 21 November 2022) for analysis and synthesis to aid exclusion and selection. A total of 1913 duplicate articles were found; after a thorough check was carried out, the duplicates were removed. The title, abstracts, and keywords of the remaining 766 articles were screened, which led to the exclusion of an additional 600 articles. The keywords used in this stage of the screening were construction, construction workers, and construction industry. Lastly, full-text screening was conducted on the remaining 66 articles, and an additional 15 studies were removed because they were either conference or review papers. Therefore, a total of 51 peer-reviewed journal articles made the final list of articles included in the review. This number is more than enough when compared to other systematic reviews of the literature in the field of mental health in the construction industry [27,30]. Figure 1 shows a summary of the article selection phases.

### 2.2. Phase 2—Prefabricated Construction and Health and Safety

The same strategy was used in Phase 2. This phase of the search was focused on identifying the general health and safety benefits of prefabricated construction to construction workers, management, and the industry at large.

#### 2.2.1. Literature Search Strategies and Terms

The keywords for the search were combined with the Boolean strings “AND” and “OR”) as: (“prefabricated construction” OR “prefabricated building” OR “modular building” OR “modular construction” OR “offsite manufacturing” OR “off-site manufacturing” OR “pre-assembly” OR “preassembly” OR “industrialized building system*” OR “industrialized construction” OR “modular assembly” OR “offsite production” OR “off-site production” OR “precast construction” OR “precast concrete construction” OR “industrialised construction*” OR “industrialized construction*” OR “industrialised construction method*” OR “industrialized construction method*” OR “panelized” OR “panellised” OR “offsite modular construction”) AND (“construction industr*” OR “aec” OR “construction sector” OR “building construction*” OR “Architectur* firm”) AND (“health and safety” OR “health & safety” OR “health” OR “safety” OR “hse” OR “well-being” OR “wellbeing”). The keywords were carefully selected by the experienced research team to include possible alternate references for this phase of the study: prefabricated construction and the health and safety of construction workers. A total of 4254 articles were obtained from the databases, with Scopus leading the pack with 2803, followed by Web of Science with 1348 and PubMed with 103.

#### 2.2.2. Exclusion and Inclusion Criteria

Exclusion and inclusion criteria set for eligibility to form a part of the review materials were set. Only the articles that met all the following inclusion criteria were included in the review:

1. Articles that studied the health and safety problems in the context of prefabricated construction;
2. Articles that based their studies on empirical qualitative and quantitative data and not data collected with pilot studies. Where pilot studies were considered, such studies must have conducted full-scale data collection and been included in the whole analysis and findings;
3. Studies based on scoping review, systematic review, and any other forms of reviews of the literature were excluded;
4. Articles must have been published in English. Articles published in other languages other than English but with official English versions available were included;
5. Articles published in peer-reviewed journals were included. Other articles, such as conference papers, book chapters, reviews, white papers, etc., were excluded even in cases where they met other inclusion requirements.

#### 2.2.3. Data Extraction and Final Article Selection

**Figure 2:**
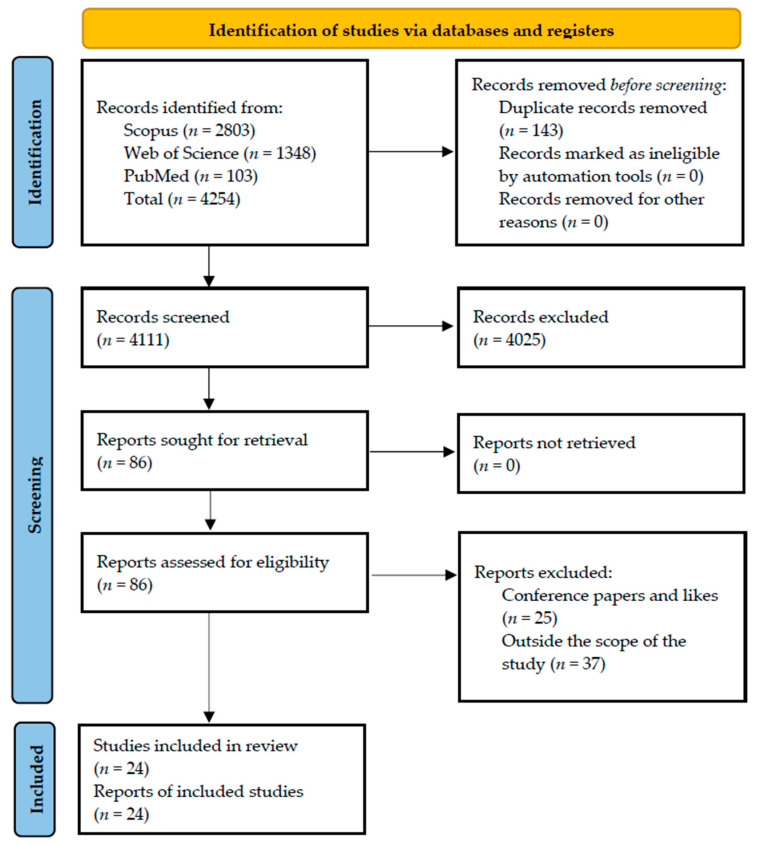
Flowchart of the literature search and selection strategy for prefabricated construction and health and safety.

To speed up the process of synthesis and analysis, https://www.rayyan.ai/ (accessed on 21 November 2022) was used again as with the first phase. A total of 143 duplicate articles were found and removed after a thorough check was carried out. The title, abstracts, and keywords of the remaining 4111 articles were screened, and an additional 4025 articles were excluded. The keywords used in screening the abstracts were prefabricated construction, modular construction, off-site, offsite, preassembly, industrialised building, panel, health, safety, and health and safety. Finally, full-text screening was conducted on the remaining 86 articles, which led to the elimination of 37 studies. Thereafter, 25 more articles were removed because they were either conference papers or review journal articles. Therefore, a total of 24 peer-reviewed journal articles made the final list of articles included in Phase 2. Figure 2 shows a summary of the article selection phases.

## 3. Results

### 3.1. Overview of the Articles Selected on Mental Health Stressors

The 51 peer-reviewed journal articles that made the final selection cover causal factors or stressors of mental health among construction workers and various intervention programs that had been adopted to address it. The earliest article [31] was published in 1993, and it was the only one from the 20th century. The remaining 50 were published between 2000 to 2022, which shows that the issue of mental health of construction workers began to gain more attention in the 21st century. Most of the articles focused on the Australian construction industry with 14 (27.45%), followed by the US and the UK with 7 (13.73%) and 5 (9.80%) respectively. Four (7.84%) of the articles were from South Africa, while the remaining 21 (41.18%) focused on other countries, including China, Ghana, India, the Netherlands, Pakistan, Nepal, Singapore, and Spain. It is therefore safe to conclude that Australia leads the research on mental health in the construction industry.

Regarding the population of the studies, 13 (25.49%) of the studies focused on all categories of construction workers, whether blue-collar or white-collar, with no distinction in terms of age, gender, or role types, be they site-based or off-site (administrative workers). Seven studies (13.73%) exclusively focused on construction professionals of all genders and ages such as architects, engineers, quantity surveyors, etc., while 5 (9.80%) based their studies only on male site workers like tradespeople and artisans. Another 4 (7.84%) studies had young construction workers as their study population, while the remaining 22 (43.14%) studied mental health and/or intervention programs, on-site supervisors, site managers, and migrant construction workers. The focus of 34 (66.67%) of those studies were on causes of poor mental health among construction workers, 12 (23.53%) were on various intervention programs designed and implemented, and the remaining 5 (9.80%) combined both stressor sources and mitigation programs.

### 3.2. Mental Health Stressors of Construction Workers

The review of the articles revealed the stressors of mental health of construction workersto be largely due to the nature of the construction industry, which influences the reaction of management/organisation and the workers engaged in the industry. This informed the classification of stressors into three (3) overarching groups: industry-related, management/organisational, and personal.

#### 3.2.1. Industry-related Stressors

These stressors breed and negatively impact both organisational and personal stressors because of their independent nature, that is, they are borne out of the nature of the activities in construction processes or are embedded in the construction business environment. These stressors dictate the level of physical, mental, technological, and other requirements of workers to fulfil their workplace obligations. Excessive work pressure [6,31,32,33], which leads to work overload [31], is a function of the heterogeneity of every project in the industry. The uniqueness of every project and the difference in every project location erodes the chances of having an absolute standardised building procedure, and this leads to ever-changing working conditions. The implication of this is that technical instructions in the construction industry are continually amended to suit every project [12] with an increased chance of experiencing ambiguity in work-related instructions and procedures [31]. Another implication of time pressure on construction workers is long working hours [1,6,32,33,34,35] which plays a major role in the poor work–life balance of construction workers [1,6,36,37,38]. 

At the end of every project, which is mostly executed at the request of a one-off client, most workers are at risk of being redundant and being laid off if the construction company fails to secure another contract. This leads to job insecurity, which has been cited as a stressor linked with the business nature of the construction industry [6,13,31,33,34,39,40,41]. Other stressors that are attributed to the nature of the industry include social isolation [36], bodily or musculoskeletal pain [3,42], physical injuries from site accidents [4,42,43,44,45], mental health stigmatisation [40], job cognitive demand [12,13,46,47,48,49], poor physical working conditions [33], increased work speed [12,13], and fatigue [50].

#### 3.2.2. Management/Organisational Stressors

These are stressors that emanate from management responses to the impacts of industry-related stressors. These stressors are negatively impacted by industry-related stressors while they, in turn, negatively impact personal stressors. Interpersonal conflicts [34], for example, could arise from improper communication of new and ever-changing instructions and ideas, which is caused by the dynamic nature of construction products. These stressors are either suppressed through proper organisational welfare support for workers or they are more pronounced through a lack of adequate support mechanisms. Stressors categorised in this group include interpersonal conflicts [48,50], inadequate work resources/facilities [33], criticism, work overload [31,32], unclear directions and instructions from management or poor communication [10,33], unfavourable shift rosters [51], poor workers support mechanisms [49], technological overload [31], lack of task autonomy [52], exclusion from decision-making [12], and poor feedback mechanisms [33].

#### 3.2.3. Personal Stressors

These are stressors developed in response to the negative impacts of both industry-related and organisational groups of stressors. Dealing with work pressure and maintaining conflict-free work relationships with colleagues, junior and senior alike, depends largely on the age of a worker [5,13,21,48,51,53,54]. The level of physical pain and stress endured by a worker is also a function of the worker’s age and gender [53]. Job insecurity caused by the fluctuation in job supply in the construction industry [31,33,34,39,41] is largely due to the industry’s characteristics and is a major source of workers’ financial challenges [4,6,34,39]. Work pressure, long working hours, and unfavourable shift rosters, as well as other unfavourable management decisions in a bid to meet contractual targets, are the main causes of the poor work–life balance of construction workers [1,36,37,55]. Stressors grouped under the personal classification include age discrimination [48,56], marital status [51], gender discrimination and [32,36] harassment [32], lack of opportunities for further training and career development [12], and financial difficulties [4,34] which usually result in low socioeconomic status among construction workers. The construction workplace attracts workers from different cultural backgrounds, and this has promoted culturally motivated stressors, such as language barriers, cultural values conflicts, and racial discrimination [33]. While it may seem reasonable to categorise cultural stressors separately, however, culture and religion have been described as important domains that significantly influence the overall personality of an individual [57]; hence, the classification under the personal stressors group.

**Figure 3:**
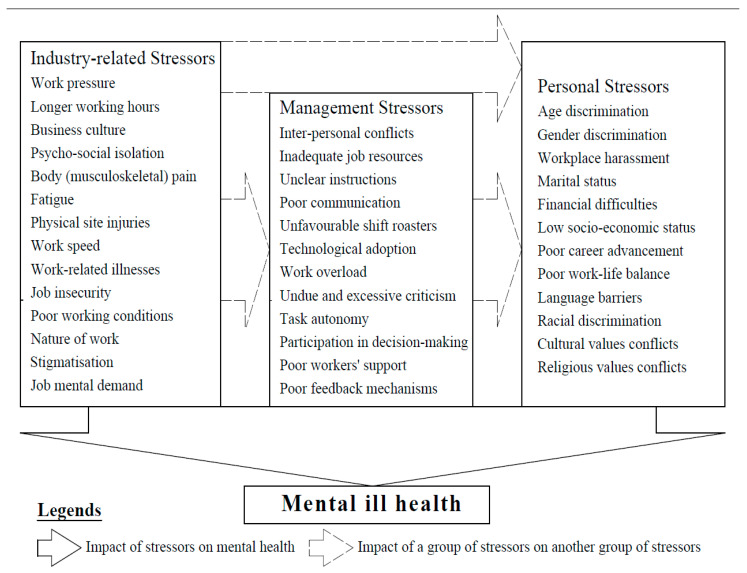
Conceptual Framework for Mental Stressors in the Construction Industry.

The stressors of mental health, as reviewed from the literature, are shown in Table 1 under their groups. Figure 3 shows the relationships between the stressor classification.

#### 3.2.4. Preventive Measures of Poor Mental Health among Construction Workers

Ten out of the 17 studies which focused on mental health intervention programs were based on the Australian construction industry, which alludes to the leading role taken by the country on the issue of mental health. The concepts of self-support, situational support, and work support were proposed by Love et al. [8] as a means of promoting good mental health among construction professionals in both consulting and contracting practices. The success of an Australian government-backed multi-modal community-based suicide prevention program, MATES in Construction, was evaluated by Gullestrup et al. [7]. It was revealed that the program enjoyed strong support from construction workers and organisations alike. However, King et al. [5,60] revealed that response and amenability to suicide and mental health literacy under the program differ by age group. 

Older workers were found to exhibit better suicide prevention literacy while the younger ones believed more in the role played by favourable workplace conditions in ameliorating the workers’ mental health conditions. The effectiveness of these awareness and literacy programs in curbing poor health behaviours like smoking and poor nutrition among workers was described as limited, as these behaviours were believed to be enhanced by the (physical and psychological) work environment rather than personal traits [1]. Long-term positive results from the training were, however, recorded in all categories of workers [61], and this was evidenced in the significant increase in demand for case management from MATES [62]. 

Another program is the Life Care Skills Program organised by “Incolink” (a joint enterprise of employer associations and industry unions in the commercial building, construction, and civil allied industries in the Australian States of Victoria and Tasmania (https://www.incolink.org.au) (accessed on 25 November 2022), which was largely reported to be somewhat successful in educating the targeted workers and engaging in outreach services to workers isolated in remote work sites [63]. 

To mitigate poor mental health among construction workers in the UK, Ajayi et al. [64] proposed a change in the management practice of the industry by setting realistic goals in terms of budget and timelines, improving communication between design and construction teams, and promoting collaboration and teamwork. Strategies to enhance employee morale and engagement, such as celebrating their success, giving constructive feedback, and promoting their interest were encouraged by Nwaogu, et al. [65]. They also recommended moves to improve interpersonal relationships by swiftly resolving workplace conflicts and promoting cordial relationships between workers and supervisors. 

While Ahmed, et al. [34] reiterate the importance of education and training for workers to enhance their mental health, Milner, et al. [66] deploy electronic intervention to discourage stigma and suicide ideation among workers. The study revealed the reluctance of workers to discuss their mental health issues, which corroborates the findings of Chapman, et al. [54] on the unwillingness of construction workers to seek help. 

The themes of most of these programs revolve around propagating mental health literacy and creating awareness of the available off-work support for construction workers. These programs are targeted at eliminating management/organisational and personal stressors. None of the intervention programs aims to eliminate the industry-related stressors which influence other stressors. Strategies to enhance the physical and psychological work environment should be embraced to make meaningful progress in improving the mental health of construction workers.

### 3.3. Prefabricated Construction

The concept of prefabrication is not new in the construction industry. The process is sometimes interchangeably referred to by other names, such as offsite construction, industrialised construction [18], offsite production [16], industrialised building system, and offsite manufacturing [11,67]. It is a method of construction in which construction is executed through the controlled offsite production of modules or components from various building materials and systems to a greater degree of finish, usually in a controlled environment, before they are transported and assembled on their permanent spot on the construction site. The prominent feature of this method is the offsite manufacturing of building components before final installation on-site. A major advantage of prefabrication over the traditional construction method is its enhancement of construction process standardisation [9], which is the process of developing templates for building components that are sometimes time-consuming, labour-intensive, and repetitive in nature for easier and faster construction in large quantities. The standardisation of construction processes positively influences cost, time, quality, and operational benefits [22]. 

Prefabrication allows for a drastic reduction in on-site activities and, by implication, reduces workers’ exposure time to inclement weather conditions [14]. Less exposure to weather conditions guarantees better control over the health and safety of workers [11]. Improved health and safety record is expected to increase job satisfaction and attract more workers to the industry.

#### Health and Safety Performance of Prefabricated Construction

The concern of back pain problems among construction workers was addressed by proposing the adoption of user-centred design aid devices in prefabricated construction, such as drill and screw guns with tripod stands for work at height; screw guns with cradles for work that involves bending and awkward postures; and lifting and carrying aid devices such as pallet jacks for lifting, carrying, and moving building panels during installation [18]. Additionally, the incorporation of ergonomic considerations in prefabricated construction designs was also recommended for reducing ergonomic hazards to construction workers [19]. The importance of design in eliminating ergonomic hazards was also established by Nussbaum, et al. [68] as well as the development of a construction management plan that eases the stacking of building panels and/or modules, location on site, delivery sequence, construction task planning, and scheduling. Furthermore, the lighter the building panels/modules are, the lesser the chances of exposing workers to physical pain [69]. Finally, where the manual carrying of prefabricated components cannot be avoided, the inclusion of an additional worker above the nominal number to a team of panel/module erectors becomes necessary. This was found to significantly reduce physical health and safety risks during the installation processes [69]. 

However, workers’ productivity and their health and safety improve due to the relocation of major parts of construction from the open, harsh, and unpredictable weather to more conducive manufacturing plants and/or factories [11,14,20]. The risks of incidents, near-misses, and other health and safety challenges could, therefore, become drastically minimised because of the reduction in on-site trade overlap [70] as health and safety threats become easier to identify and control through improved cooperation and safety consciousness among workers in prefabrication settings [71]. This translates to an increase in off-site personnel and operations and a corresponding decrease in on-site personnel and operations in the construction phase of prefabricated construction. Consequently, the frequency of dangerous tasks, such as working at height, exposed edges, etc., could be reduced as well as the number of workers involved in such operations. Where the dangers cannot be considerably reduced [72], identifying them at the early stage is easier, and proper control measures are put in place to protect workers [73]. In addition to providing ample space for injury-free work, prefabrication also facilitates cleaner construction sites [20]. This shields the workers from noxious materials that could lead to chronic health risks. Risky on-site activities that can lead to incidents like falling from heights, being trapped in-between spaces or equipment, and other related site incidents, are reduced through the prefabrication of building components, panels, or modules in the comfort of factories. Therefore, the use of prefabrication reduces ergonomic hazards for workers because of the decline in physically demanding activities [18]. On a broader note, prefabrication enhances the safety of the work environment and lower incidents and injury rates among construction workers because of less time spent on-site in comparison with the traditional method. This was the conclusion of Rubio-Romero et al. [74], which links the frequency and rate of site injuries and incidents to the length of on-site time against the whole project duration. While the method is not completely incident-free, it can drastically weaken the possibility of the occurrence of site accidents through cleaner [20] and less weather-dependent construction [11,14]. Table 2 summarises the health and safety benefits of prefabricated construction.

## 4. Discussion: Prefabricated Construction as a Tool for Good Mental Health

To date, all mental health and suicide prevention intervention programs that have been established and implemented in the construction industry have been targeted at stressors of management/organisational and personal nature [5,63]. Although there were suggestions on addressing the nature, culture, and working conditions of the industry to achieve meaningful progress in improving workers’ mental health [1], no study had proposed how this can be achieved. Although the intervention programs educate all construction workers on the need to seek help to manage poor mental health [54], this does not prevent the workers’ exposure to health stressors [66]. Instead, they tend to provide guidance on how to manage the stressors and pain that appear to have been accepted as the norm in the industry. However, documented advantages of prefabrication over the traditional construction method in the literature indicate that the method has the potential to positively impact the mental health of workers by drastically changing the nature of construction operations and, consequently, reducing the impact of work-related stressors.

Adopting the prefabrication method for construction projects can improve construction quality, reduce labour inefficiency, and increase efficiency through the standardisation of processes [9]. This reduces the overall time spent on construction work and enhances the work–life balance of workers. With a standardised approach to construction, ambiguity in roles and interpersonal conflicts associated with it, such as bullying and criticism, are reduced.

Although the project design time is usually shorter in traditional construction than in prefabricated construction, the overall project duration of prefabrication is mostly shorter due to the possibility of simultaneous site preparation and off-site building component and/or module manufacturing [11]. The synchronous off-site manufacture and on-site pre-installation activities, under normal circumstances, reduce workers’ congestion on the project site [70,77] through the reduction in on-site trade overlap. With a reduction in project duration and unambiguous design and construction information, work pressure and all other associated work-related stressors such as work overload, fatigue, long work hours, and unsafe work speed could be reduced. These primary or industry-related stressors directly influence management and personal coping mechanisms. Hence, prefabrication being capable of delivering a quality project in a shorter time while increasing productivity could positively impact the mental health of workers by potentially weakening the impacts of industry-related stressors that are the causes of other stressors.

Health and safety risks are easier to identify at an early stage of prefabricated construction and, consequently, facilitate a safer construction than the traditional approach [75]. Relocating a considerable phase of construction activities to a controlled factory environment, where safety may be better managed, could reduce the frequency of dangerous tasks undertaken on project sites. The residual on-site safety hazards could be efficiently managed due to the reduction in congestion of workers and less trade overlap on prefabricated construction sites [14]. Prefabrication also promotes less physically demanding work and more ergonomically compliant mechanical tools and equipment [18]. This reduces the development of body pains and musculoskeletal disorders, both of which have been strongly linked with adjustment disorders (anxiety and depression) in workers [3]. Moreover, less physical, and manual work will attract more women into construction and improve the male–female ratio of workers in the industry. A balanced male–female workers ratio will reduce the industry’s masculine image and could discourage gender-based harassment and discrimination. Reduction in all forms of discrimination will also promote healthy interpersonal relationships and a better psychological work environment.

In addition to a better psychological work environment, prefabricated construction also enhances cleaner construction with ease of housekeeping [20] because of the disaggregation of tasks through standardisation. This, therefore, ensures that workers carry out their duties in a good physical work environment and reduces the chances of safety incidents and injuries occurrence. Pain-inducing traditional construction tasks are made less painful, and bodily and/or musculoskeletal pains are considerably reduced through prefabrication.

**Figure 4:**
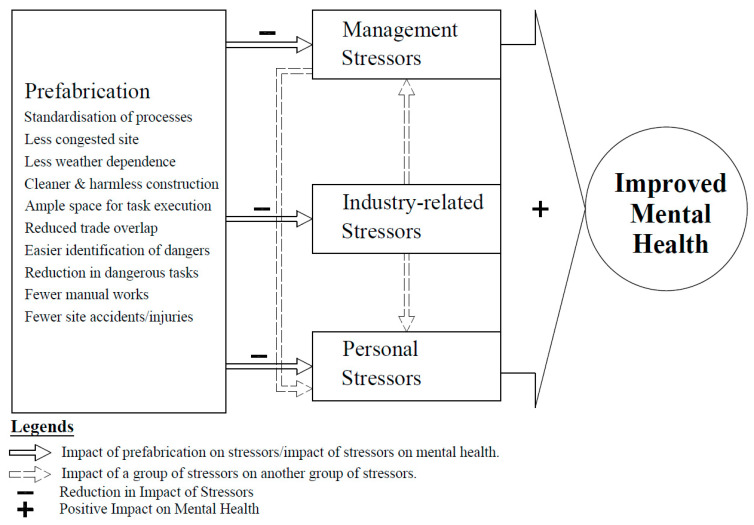
Conceptual Framework on the Influence of Prefabrication on the Mental Health of Construction Workers.

While the demand for construction services is completely outside the control of the construction industry, the method by which the service is provided, which induces mental health challenges in workers, can, however, be controlled and managed without compromising quality and time performance through the implementation of prefabricated construction. Figure 4 shows the conceptual relationship between the benefits of prefabricated construction, stressors, and the mental health of workers.

Unlike the traditional construction method, prefabrication ensures that the processes of construction are standardised [9]. Standardisation ensures the simplicity of task information and reduces ambiguity without compromising the uniqueness of every project. The elimination of ambiguity enhances project communication which, in turn, positively impacts interpersonal relationships, improves feedback mechanisms, and reduces the impact of language barriers. Excessive explanation, which leads to communication difficulties among construction team members of different first languages, can be significantly reduced if the tasks in projects are achieved through extensive, regular, and repetitive manufacturing of panels, modules, or units. Clarity of information enhances younger and new workers’ understanding of instructions, and this can reduce frequent mistakes and criticism. Standardisation also enhances mechanisation, and this reduces manual tasks [18], body pain, and can encourage the presence of more female workers. Furthermore, standardisation requires early freezing of designs, and this can drastically reduce variation requests and rework. A better project resources plan can be developed, thereby enhancing the chances of providing workers with adequate construction resources and facilities.

Prefabrication enhances workers’ productivity [75,76]. Increased productivity reduces workload and work pressure. Furthermore, the reduction in the dependence on weather [14] ensures a reduction in both on-site and overall project duration. In addition to the better project time management benefit of prefabrication, the prospect of reducing time pressure and improving work–life balance and, consequently, reducing workers’ health stressors, is enhanced. Less work-related stress could be the much-needed catalyst for workers to undertake further learning for their personal and career development. This can enhance their skills and could ensure job security. With career development comes promotion, task autonomy, and decision-making roles. These can enhance workers’ socio-economic status.

**Figure 5:**
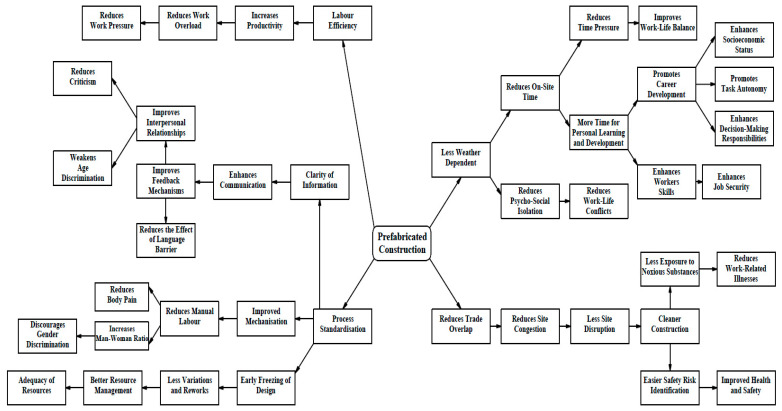
Impacts of Prefabrication on Construction Workers’ Mental Health Stressors.

Due to the reduction in trade overlap, the number of workers on-site during the installation of building components is reduced to a manageable minimum. This reduces site disruptions, promotes cleaner construction, and reduces the exposure of workers to noxious substances since most of the work is performed in a controlled factory environment. Work-related illness is, therefore, reduced and the chances of falls, being struck by falling objects, or stepping on live wires are reduced. Figure 5 shows how prefabricated construction can positively impact the mental health stressors of construction workers.

## 5. Limitations

Restricting the language of the articles selected for this study to English only is a key limitation. Journal articles with strong and different perspectives on the issues addressed could have been excluded on the grounds of the language of publication or lack of translated copies. However, the use of multiple database searches and the coverage of the selected databases ensure that qualified journal articles published in English, the language of the study, were included in the screening processes.

While the outcome of the review indicates a potential positive impact of prefabrication on improved mental health for construction workers, this position remains a subjective view that can only be tested and confirmed through an empirical data-driven study. 

## 6. Conclusions

The consequences of the declining state of the mental health of construction workers are felt beyond the construction industry. The industry loses its active workers to mental health challenges and suicide, thereby reducing its productivity, contribution to economic growth, and overall workers’ living standards. Past studies have identified stressors and prescribed measures to confront mental health challenges. However, the impacts of these intervention programs seem to be limited. This is attributed to the inability of the measures to address industry-related stressors, which this study revealed as the primary sources of mental health challenges. This study, therefore, categorised the stressors into three groups and examined the relationship between the groups.

This study also reveals the potential of prefabricated construction to reduce or eliminate the impact of industry-related stressors. This is largely due to the capability of the method to enhance the physical safety of workers, promote a good physical and psychological work environment, reduce physical pains and fatigue, and promote a healthy work–life balance among construction workers.

## 7. Recommendation

This study classified mental health stressors into Industry-related, Management, and Personal stressors. A theoretical framework was also developed to indicate the probable influence of industry-related stressors over management and personal stressors. Being the theoretical causal factors for the other two stressor groups, reducing the impact of industry-related stressors could reduce the impacts of management and personal stressors on workers. 

The health and safety benefits of prefabricated construction were also analysed from the literature, and these benefits were linked with reducing work-related stressors and improving the mental health of workers. The reported findings are conceptual in nature. Therefore, the practical impact of prefabrication on mental health stressors among construction workers should be confirmed through the collection and analysis of empirical data. Further study can be developed to compare the degree of experience of mental health stressors of workers engaged in traditional and prefabricated construction projects.

This study proposed the possibility of addressing mental health issues from the lens of the construction methods that are in use in the industry. Future research could yield measures to tackle mental health stressors and conditions from a proactive standpoint rather than from a reactive perspective by dealing with the stressors embedded in the methods of construction.

## Figures and Tables

**Table 1:** Summary of Mental Health Stressors among Construction Workers.

SN	Mental Health Stressors	Reference(s)
A	Industry-related Stressors	
1	Work pressure	[6,31,32,33]
2	Long working hours	[1,6,32,33,34,35]
3	Psycho–social isolation	[36]
4	Business culture	[6,13,31,33,34,39,40,41]
5	Bodily or musculoskeletal pain	[3,42]
6	Physical injuries from site accidents	[4,42,43,44,45]
7	Fatigue	[50]
8	Unhealthy increase in work speed	[12,13]
9	Work-related physical illness	[4,44]
10	Job insecurity	[31,33,34,39,41]
11	Poor working condition	[33]
12	Nature of work	[34,39]
13	The stigma attached to mental health	[40]
14	Job mental (cognitive) demand	[12,13,46,47,48,49]
B	Management/Organisational Stressors	
1	Interpersonal conflicts	[34,48,50]
2	Inadequate job resources	[33]
3	Unclear directions from supervisors and management	[10,31,33]
4	Poor communication	[33,58]
5	Unfavourable shift rosters	[51]
6	Technology overload, e.g., BIM, drones, etc.	[31]
7	Work overload	[31,32]
8	Undue and excessive criticism	[32]
9	Lack of task autonomy	[52]
10	Lack of participation in decision-making	[12]
11	Poor workers’ support mechanism	[49]
12	Poor feedback mechanism	[33]
C	Personal Stressors	
1	Age discrimination	[48]
2	Gender discrimination	[32]
3	Workplace harassment	[32,36]
4	Marital status	[51,53]
5	Financial difficulties	[4,34]
6	Low socio–economic status	[41]
7	Opportunities for further learning	[12]
8	Poor work–life balance	[1,6,36,37]
9	Language barriers	[33]
10	Racial discrimination	[33,58]
11	Cultural/Religious values conflicts	[33,58,59]

**Table 2:** Health and Safety Benefits of Prefabricated Construction.

SN	Benefits of Prefabrication	Reference(s)
1	Construction process standardisation	[9]
2	Safety risks become easier to identify and control	[75]
3	Reduction in trade overlap and site congestion	[70]
4	Reduction in people working in dangerous positions	[14]
5	Reduction in the frequency of dangerous works on site	[14]
6	Cleaner construction and less exposure to harm	[20]
7	Ample space for safe preassembly of components	[20]
8	Reduction in physically demanding on-site tasks	[18]
9	Less time spent on site and exposure to weather	[14]
10	Reduced chances of occurrence of falls, struck-by, etc.	[18,76]

## Data Availability

Not applicable.
